# On soft submaximal spaces

**DOI:** 10.1016/j.heliyon.2022.e10574

**Published:** 2022-09-13

**Authors:** Samer Al Ghour, Zanyar A. Ameen

**Affiliations:** a Department of Mathematics and Statistics, Jordan University of Science and Technology, Irbid 22110, Jordan; b Department of Mathematics, College of Science, University of Duhok, Duhok 42001, Iraq

**Keywords:** Soft submaximal space, Soft locally closed set, Soft dense set

## Abstract

In the present paper, detailed properties of soft submaximal topological spaces have been discussed. It starts by examining how well soft submaximal spaces behave when subjected to some operations, such as: taking soft subspaces, soft products, soft topological sum and images/preimages under certain soft mappings. Furthermore, several characterizations of soft submaximal spaces are obtained with respect to various types of soft subsets of soft topological spaces and their simple extensions.

## 1 Introduction

The area of topology which focuses on the fundamental set-theoretic concepts and procedures utilized in topology is known as general topology. It is the foundation of other branches of topology, including geometric topology, algebraic topology, and differential topology. The notion of submaximal spaces was introduced by Bourbaki [15] as a tool to study the topological spaces that do not admit a larger topology with the same semi-regularization. Such spaces were considered as a significant topic in both topological space and topological group, (see [10], [17]). Soft topology, particularly merges soft set theory and topology, is also a field of topology. It is driven by the basic assumptions of classic topological space and focuses on the set of all soft sets. A parameterized family of sets is known as a soft set. It was proposed by Molodtsov [28] in 1999 to concern with uncertainties. Shabir and Naz's [30] contributions were especially important in establishing the area of soft topology. Later several subclasses of soft topological spaces were suggested, including soft separation axioms [1], [3], [14], soft separable spaces [14], soft connected [25], soft compact [13], [32], soft paracompact [25], soft extremally disconnected [11], soft J-spaces [27], and soft (nearly) Menger spaces [4], [24]. Furthermore, soft bioperators on soft topological spaces have been studied in [12]. Despite the fact that many studies followed their instructions and many notions developed in soft environments, substantial contributions can also be provided. The class of soft submaximal spaces was defined by Ilango and Ravindran [21], but not much study was done to further investigate soft submaximal spaces. The topic is crucial for considering the maximal element in the set of all soft topologies (see [2]) or soft topological groups on a common universe, and for extending many other different results done in [10]. Hence, we develop the properties of soft submaximal spaces and obtain several of their characterizations.

## 2 Preliminaries

Given a (discourse) set *Z* and an attribute set Λ. A soft set over (or in) *Z* is a pair (Y,Λ)={(e,Y(e)):e∈E} such that $Y:\Lambda \to 2^{Z}$ is a set-valued mapping. Denote by $P_{\Lambda }(Z)$ the set of all soft subsets in *Z*. A soft set (Y,Λ) in *Z* is called a soft element [29], denoted by ({z},Λ), if (Y,Λ)={z} for all e∈Λ. A soft set (Y,Λ) in *Z* is called soft point [7], denoted by z(e), if Y(e)={z} and $Y(e^{\prime })=\emptyset$ for each $e^{\prime }\neq e$, $e^{\prime }\in \Lambda$. The notation $z(e)\overset{˜}{\in }(Y,\Lambda )$ implies that z∈Y(e). Two soft points $z(e),y(e^{\prime })$ are said to be distinct if either z≠y or $e\neq e^{\prime }$. The soft sets (Y,Λ),(X,Λ) over *Z* are said to be disjoint if $(Y,\Lambda )\overset{˜}{\cap }(X,\Lambda )=\overset{˜}{\Phi }$. By a singleton soft set we mean set that includes one soft point. The complement of (Y,Λ) is a soft set ${\left(Y,\Lambda \right)}^{c})$ such that $Y^{c}:E\to 2^{Z}$ is defined by $Y^{c}(e)=Z﹨Y(e)$ for all e∈Λ (we may refer to (Z,Λ)﹨(Y,Λ) as the complement of (Y,Λ)). A null soft set $\overset{˜}{\Phi }$ is a soft set (Y,Λ) for which Y(e)=∅ for any e∈Λ. An absolute soft set $\overset{˜}{Z}$ is a soft set (Y,Λ) for which Y(e)=Z for any e∈Λ. Evidently, ${\overset{˜}{\Phi }}^{c}=\overset{˜}{Z}$ and ${\overset{˜}{Z}}^{c}=\overset{˜}{\Phi }$. A soft set $\left(X,\Lambda_{1}\right)$ is called a subset of $\left(Y,\Lambda_{2}\right)$ (denoted by $\left(X,\Lambda_{1})\overset{˜}{\subseteq }(Y,\Lambda_{2}\right)$, [26]) whenever $\Lambda_{1}\subseteq \Lambda_{2}$ and X(e)⊆Y(e) for all $e\in \Lambda_{1}$. Clearly, $\left(X,\Lambda_{1})=(Y,\Lambda_{2}\right)$ whenever $\left(X,\Lambda_{1})\overset{˜}{\subseteq }(Y,\Lambda_{2}\right)$ and $\left(Y,\Lambda_{2})\overset{˜}{\subseteq }(X,\Lambda_{1}\right)$. The union of soft sets (R,Λ),(S,Λ) is defined by $\left(F,\Lambda )=(R,\Lambda )\overset{˜}{\cup }(S,\Lambda \right)$, where F(e)=R(e)∪S(e) for every e∈Λ, and the intersection of soft sets (R,Λ),(S,Λ) is defined by $\left(F,\Lambda )=(R,\Lambda )\overset{˜}{\cap }(S,\Lambda \right)$, where F(e)=R(e)∩S(e) for every e∈Λ, (see, [6], [31]).

Definition 2.1 [18], [30] A soft topology on *Z* is a collection T of $P_{\Lambda }(Z)$ if T meets the following conditions:

(1) $\overset{˜}{\Phi },\overset{˜}{Z}\overset{˜}{\in }T$;
(2) $(B_{1},\Lambda )\overset{˜}{\cap }(B_{2},\Lambda )\overset{˜}{\in }T$ for each $(B_{1},\Lambda ),(B_{2},\Lambda )\overset{˜}{\in }T$; and
(3) ${\overset{˜}{⋃}}_{i\in I}(B_{i},\Lambda )\overset{˜}{\in }T$ for any $\{(B_{i},\Lambda ):i\in I\}\overset{˜}{\subseteq }T$.

Terminologically, we call (Z,T,Λ) a soft topological space on *Z*. The members of T are known as soft T-open sets (or shortly soft open sets when there is no confusion), and their complements are known as soft T-closed sets (or shortly soft closed sets).

In the next, we refer to a soft topological space as (Z,T,Λ).

Definition 2.2 [16] Given a soft topology T. A soft base for T is a subcollection B⊆T such that elements of T are unions of elements of B.

Definition 2.3 [30] Let (Z,T,Λ) be a soft topological space and let $\left(Y,\Lambda )\overset{˜}{\in }P_{\Lambda }(Z\right)$. A soft relative topology on *Y* is defined by

$$T_{Y}:=\{(G,\Lambda )\overset{˜}{\cap }(Y,\Lambda ):(G,\Lambda )\overset{˜}{\in }T\}.$$

The triple $\left(Y,T_{Y},\Lambda \right)$ is called a soft subspace of (Z,T,Λ).

Definition 2.4 [30] Let (Z,T,Λ) be a soft topological space and let $\left(R,\Lambda )\overset{˜}{\in }P_{\Lambda }(Z\right)$. The soft interior of (R,Λ) is

$${\mathrm{Int}}_{T}((R,\Lambda )):=\overset{˜}{⋃}\{(G,\Lambda ):(G,\Lambda )\in T,(G,\Lambda )\overset{˜}{\subseteq }(R,\Lambda )\}.$$

The soft closure of (R,Λ) is

$${\mathrm{Cl}}_{T}((R,\Lambda ))):=\overset{˜}{⋂}\{(G,\Lambda ):{\left(G,\Lambda \right)}^{c}\in T,(G,\Lambda )\overset{˜}{⊇}(R,\Lambda )\}.$$

If $\left(X,T_{X},\Lambda \right)$ is a soft subspace, the soft interior and soft closure of a soft (R,Λ) in $\left(X,T_{X},\Lambda \right)$ are denoted by ${\mathrm{Cl}}_{X}((R,\Lambda )))$ and ${\mathrm{Int}}_{X}((R,\Lambda ))$, respectively. The soft boundary of (R,Λ) is defined by $\mathrm{Bd}((R,\Lambda ))=\mathrm{Cl}((R,\Lambda ))\overset{˜}{\cap }\mathrm{Cl}({\left(R,\Lambda \right)}^{c})$.

Definition 2.5 [20] Let (Z,T,Λ) be a soft topological space and let $\left(R,\Lambda )\overset{˜}{\in }P_{\Lambda }(Z\right)$. A soft point $z(e)\overset{˜}{\in }\overset{˜}{Z}$ is called a soft limit point of (R,Λ) if

$$(R,\Lambda )\overset{˜}{⋂}(G,\Lambda )﹨\{z(e)\}\neq \overset{˜}{\Phi }$$

for each (G,Λ)∈T with $z(e)\overset{˜}{\in }(G,\Lambda )$.

Lemma 2.6 *[20]* *Let* (Z,T,Λ) *be a soft topological space and let* $\left(R,\Lambda )\overset{˜}{\in }P_{\Lambda }(Z\right)$ *. Then*

$${\mathrm{Int}}_{T}({\left(Y,\Lambda \right)}^{c})={\left({\mathrm{Cl}}_{T}((Y,\Lambda ))\right)}^{c}\;\text{and}\;{\mathrm{Cl}}_{T}({\left(Y,\Lambda \right)}^{c})={\left({\mathrm{Int}}_{T}((Y,\Lambda ))\right)}^{c}.$$

Definition 2.7 Let (Z,T,Λ) be a soft topological space and let $\left(X,\Lambda ),(Y,\Lambda )\overset{˜}{\in }P_{\Lambda }(Z\right)$. Then (X,Λ) is called

(1) soft regular open [19] if (X,Λ)=Int(Cl((X,Λ))),
(2) soft dense [9] in (Y,Λ) if $\left(Y,\Lambda )\overset{˜}{\subseteq }\mathrm{Cl}((X,\Lambda )\right)$,
(3) soft co-dense [9] if $\mathrm{Int}((X,\Lambda ))=\overset{˜}{\Phi }$,
(4) soft preopen [21] if $\left(X,\Lambda )\overset{˜}{\subseteq }\mathrm{Int}(\mathrm{Cl}((X,\Lambda ))\right)$,
(5) soft locally closed [23] if $\left(X,\Lambda )=(G,\Lambda )\overset{˜}{\cap }(F,\Lambda \right)$, where $(G,\Lambda )\overset{˜}{\in }T$ and ${\left(F,\Lambda \right)}^{c}\overset{˜}{\in }T$,
(6) soft co-locally closed if $\left(X,\Lambda )=(G,\Lambda )\overset{˜}{\cup }(F,\Lambda \right)$, where $(G,\Lambda )\overset{˜}{\in }T$ and ${\left(F,\Lambda \right)}^{c}\overset{˜}{\in }T$.

Definition 2.8 [22], [33] Let $\left(R,\Lambda ),(S,\Lambda^{\prime }\right)$ be soft sets, and let $g:R\to S,h:\Lambda \to \Lambda^{\prime }$ be mappings. The image of a soft set $\left(B,\Lambda )\overset{˜}{\subseteq }(R,\Lambda \right)$ under $f:(R,\Lambda )\to (S,\Lambda^{\prime })$ is a soft subset f(B,Λ)=(f(B),h(Λ)) of $\left(S,\Lambda^{\prime }\right)$ which is given by

$$f(B)(e^{\prime })=\left\{ \begin{array}{cc} \underset{e\in h^{-1}(e^{\prime })\cap \Lambda }{⋃}g\left(B(e)\right), & h^{-1}(e^{\prime })\cap \Lambda \neq \emptyset ;\\ \emptyset , & \text{ otherwise}, \end{array}\right.$$

for each $e^{\prime }\in \Lambda^{\prime }$.

The inverse image of a soft set $\left(B,\Lambda^{\prime })\overset{˜}{\subseteq }(S,\Lambda^{\prime }\right)$ under *f* is a soft subset $f^{-1}(B,\Lambda^{\prime })=(f^{-1}(B),h^{-1}(\Lambda^{\prime }))$ such that

$$(f^{-1}(B)(e)=\left\{ \begin{array}{cc} g^{-1}\left(B(h(e))\right), & h(e)\in \Lambda^{\prime };\\ \emptyset , & \text{ otherwise}, \end{array}\right.$$

for each e∈Λ.

If both mappings *g* and *h* are bijective, then *f* is bijective.

Definition 2.9 [29] A soft mapping *f* from a soft topological space (Z,T,Λ) into a soft topological space $\left(Y,T^{\prime },\Lambda^{\prime }\right)$ is called

(1) soft continuous if $f^{-1}((H,\Lambda^{\prime }))\in T$ for each $(H,\Lambda^{\prime })\in T^{\prime }$.
(2) soft open if $f((G,\Lambda ))\in T^{\prime }$ for each (G,Λ)∈T.
(3) soft homeomorphism if it is a soft open and a soft continuous bijection.

Definition 2.10 [13] Let $\left\{(Z_{i},T_{i},\Lambda ):i\in I\right\}$ be a collection of soft topological spaces with a fixed parametric set Λ. The product soft topology T on $Z=\prod_{i\in I}Z_{i}$ is the initial soft topology on *Z* generated by the family $\left\{p_{i}:i\in I\right\}$, where $p_{i}$ is the soft projection mapping from *Z* to $Z_{i}$ for i∈I.

Definition 2.11 [5] Let $\left\{(Z_{i},T_{i},\Lambda ):i\in I\right\}$ be a collection of soft topological spaces such that $Z_{i}\overset{˜}{\cap }Z_{j}=\overset{˜}{\Phi }$ for each i≠j. The soft topology T on ${\overset{˜}{⋃}}_{i\in I}{\overset{˜}{Z}}_{i}$ generated by the basis $B=\{(U,\Lambda )\overset{˜}{\subseteq }{\overset{˜}{⋃}}_{i\in I}{\overset{˜}{Z}}_{i}:(U,\Lambda )\overset{˜}{\in }T_{i}\text{ for some }i\}$ is called sum of soft topological spaces and denoted by $\left({⨁}_{i\in I}Z_{i},T,\Lambda \right)$.

## 3 Some operations on soft submaximal spaces

Definition 3.1 [21] A soft submaximal space is soft topological space (Z,T,Λ) in which each soft dense set over *Z* is soft open.

We start by showing soft submaximality is a hereditary property.

Proposition 3.2 *If*$\left(Y,T_{Y},\Lambda \right)$*is a soft subspace of a soft submaximal space*(Z,T,Λ)*, then*$\left(Y,T_{Y},\Lambda \right)$*is soft submaximal.*

Proof Let (D,Λ) be soft dense over *Y*. Then $\left(Y,\Lambda )\overset{˜}{\subseteq }\mathrm{Cl}((D,\Lambda )\right)$. But (D,Λ) $\overset{˜}{\cup }$ $\left(\overset{˜}{Z}﹨\mathrm{Cl}((D,\Lambda ))\right)$ is soft dense over *Z* and, by soft submaximality of (Z,T,Λ), $\left(D,\Lambda )\overset{˜}{\cup }(\overset{˜}{Z}﹨\mathrm{Cl}((D,\Lambda ))\right)$ is a soft open set over *Z*. Now, we have $\left(D,\Lambda )\overset{˜}{\cup }(\overset{˜}{Z}﹨\mathrm{Cl}((D,\Lambda )))\overset{˜}{⋂}(Y,\Lambda )=(D,\Lambda \right)$ and so, by Theorem 2 in [30], (D,Λ) is soft open in (Y,Λ). Hence $\left(Y,T_{Y},\Lambda \right)$ is soft submaximal. □

Proposition 3.3 *Let* $\left(Z,T,\Lambda ),(Z,T^{\prime },\Lambda \right)$ *be soft topological spaces and* $T\overset{˜}{\subset }T^{\prime }$ *. If* T *is a soft submaximal space, then* $T^{\prime }$ *is soft submaximal.*

Proof If (D,Λ) is a soft $T^{\prime }$-dense set over *Z*, then $\overset{˜}{Z}={\mathrm{Cl}}_{T}^{\prime }((D,\Lambda ))\overset{˜}{\subseteq }{\mathrm{Cl}}_{T}((D,\Lambda ))$ and so (D,Λ) is soft T-dense. But (Z,T,Λ) is soft submaximal, then (D,Λ) is soft T-open. Since $T\overset{˜}{\subset }T^{\prime }$, so (D,Λ) is soft $T^{\prime }$-open and thus $\left(Z,T^{\prime },\Lambda \right)$ is a soft submaximal space. □

Lemma 3.4 *If* $f:(Z,T,\Lambda )\to (Y,T^{\prime },\Lambda^{\prime })$ *is a soft open mapping, then*

$$f^{-1}({\mathrm{Cl}}_{T}^{\prime }((B,\Lambda^{\prime })))\overset{˜}{\subseteq }{\mathrm{Cl}}_{T}(f^{-1}((B,\Lambda^{\prime })))$$

*for each soft set* $\left(B,\Lambda^{\prime }\right)$ *over Y.*

Proof Suppose *f* is soft open. Let $(F,\Lambda^{\prime })\overset{˜}{\subseteq }\overset{˜}{Y}$ and let $z(e)\overset{˜}{\in }f^{-1}({\mathrm{Cl}}_{T}^{\prime }((F,\Lambda^{\prime })))$. Then $f(z(e))\overset{˜}{\in }{\mathrm{Cl}}_{T}^{\prime }((F,\Lambda^{\prime }))$. Assume (G,Λ) is any soft T-open set including z(e). By soft openness of *f*, f((G,Λ)) is soft $T^{\prime }$-open and so $(F,\Lambda^{\prime })\overset{˜}{\cap }f((G,\Lambda ))\neq \overset{˜}{\Phi }$. Therefore $f^{-1}((F,\Lambda^{\prime }))\overset{˜}{\cap }(G,\Lambda )\neq \overset{˜}{\Phi }$. Thus $z(e)\overset{˜}{\in }{\mathrm{Cl}}_{T}(f^{-1}((F,\Lambda^{\prime })))$. The proof is finished. □

Proposition 3.5 *If* $f:(Z,T,\Lambda )\to (Y,T^{\prime },\Lambda^{\prime })$ *is a soft open surjection and* (Z,T,Λ) *is a soft submaximal space, then* $\left(Y,T^{\prime },\Lambda^{\prime }\right)$ *is soft submaximal.*

Proof Let $\left(H,\Lambda^{\prime }\right)$ be a soft $T^{\prime }$-dense set over *Y*. By Lemma 3.4, $\overset{˜}{Z}=f^{-1}({\mathrm{Cl}}_{T}^{\prime }((H,\Lambda^{\prime })))⊑{\mathrm{Cl}}_{T}(f^{-1}((H,\Lambda^{\prime })))$ and thus $f^{-1}((H,\Lambda^{\prime }))$ is soft T-dense over *Z*. By soft submaximality of (Z,T,Λ), $f^{-1}((H,\Lambda^{\prime }))$ is soft T-open. Since *f* is soft open surjective, then $f(f^{-1}((H,\Lambda^{\prime })))=(H,\Lambda^{\prime })$ soft open over *Y*. Hence $\left(Y,T^{\prime },\Lambda^{\prime }\right)$ is soft submaximal. □

One might conjecture that the inverse image of a soft submaximal space under soft continuous is soft submaximal, but this is generally false.

Example 3.6 Let Z={1,2} and R be the set of reals. For any parametric set Λ, we respectively define soft topologies $T=\{\overset{˜}{\Phi },(\{1\},\Lambda ),(\{2\},\Lambda ),\overset{˜}{Z}\}$ and $T^{\prime }=\{\overset{˜}{\Phi },\overset{˜}{Q},\overset{˜}{P},\overset{˜}{R}\}$ on *Z* and R, where Q is the set of rationals and P is the set of irrationals. The soft mapping $f:(R,T^{\prime },\Lambda )\to (Z,T,\Lambda )$ defined by

$$f(r)=\left\{ \begin{array}{c} 1,\text{ if }r\in Q;\\ 2,\text{ if }r\in P, \end{array}\right.$$

is soft continuous and (Z,T,Λ) is soft submaximal, while $\left(R,T^{\prime },\Lambda \right)$ is not soft submaximal.

However, the following result is true:

Corollary 3.7 *Let*$f:(Z,T,\Lambda )\to (Y,T^{\prime },\Lambda^{\prime })$*be a soft homeomorphism. Then*(Z,T,Λ)*is soft submaximal if and only if*$\left(Y,T^{\prime },\Lambda^{\prime }\right)$*is soft submaximal.*

Lemma 3.8 *[13, Theorem 3.8]* *The soft projection mapping* $p_{i}:{\left(\prod Z_{i},\prod T_{i},\Lambda \right)}_{i\in I}\to (Z_{i},T_{i},\Lambda )$ *is soft open for each i.*

Proposition 3.9 *If the product soft space* ${\left(\prod Z_{i},\prod T_{i},\Lambda \right)}_{i\in I}$ *is soft submaximal, then* $\left(Z_{i},T_{i},\Lambda \right)$ *is soft submaximal for each i.*

Proof Follows from Proposition 3.5 and Lemma 3.8. □

The converse of the above proposition is not true.

Example 3.10 Let Z={0,1} and let Λ be any set parameters. Define a soft topology on *Z* by $T=\{\overset{˜}{\Phi },(\{0\},\Lambda ),\overset{˜}{Z}\}$. The soft submaximality of (Z,T,Λ) can be easily followed, but not of (Z×Z,T×T,Λ).

Proposition 3.11 *The soft topological sum* $\left({⨁}_{i\in I}Z_{i},T,\Lambda \right)$ *is soft submaximal if and only if* $\left(Z_{i},T_{i},\Lambda \right)$ *is soft submaximal for each i.*

Proof The first direction follows from Proposition 3.2.

Conversely, if (D,Λ) is soft dense over ${⨁}_{i\in I}Z_{i}$, then $(D,\Lambda )\overset{˜}{\cap }Z_{i}$ is soft dense over $Z_{i}$ for each *i*. Since $\left(Z_{i},T_{i},\Lambda \right)$ is soft submaximal, then $(D,\Lambda )\overset{˜}{\cap }Z_{i}$ is soft open over $Z_{i}$ for each *i*, which implies that $(D,\Lambda )\overset{˜}{\cap }Z_{i}$ is soft open over ${⨁}_{i\in I}Z_{i}$. Therefore $\left(D,\Lambda )={\overset{˜}{⋃}}_{i\in I}[(D,\Lambda )\overset{˜}{\cap }Z_{i}\right]$ is a soft open set over ${⨁}_{i\in I}Z_{i}$. Thus $\left({⨁}_{i\in I}Z_{i},T,\Lambda \right)$ is soft submaximal. □

Proposition 3.12 *Let* (G,Λ) *be a soft subset of a soft submaximal space* (Z,T,Λ) *. Then* (G,Λ) *is soft open if and only if* $\left(G,\Lambda )=(R,\Lambda )\overset{˜}{\cap }(D,\Lambda \right)$ *for some soft regular open* (R,Λ) *and soft dense* (D,Λ) *in* $\overset{˜}{Z}$ *.*

Proof If (G,Λ) is soft open, then $\left(G,\Lambda )\overset{˜}{\subseteq }\mathrm{Int}(\mathrm{Cl}((G,\Lambda ))\right)$ and so

$$(G,\Lambda )=\mathrm{Cl}((G,\Lambda ))﹨\mathrm{Cl}((G,\Lambda ))﹨(G,\Lambda )=\mathrm{Cl}((G,\Lambda ))\overset{˜}{\cap }[\overset{˜}{Z}﹨(\mathrm{Cl}((G,\Lambda ))﹨(G,\Lambda ))]=\mathrm{Int}(\mathrm{Cl}((G,\Lambda )))\overset{˜}{\cap }[(G,\Lambda )\overset{˜}{\cup }(\overset{˜}{Z}﹨\mathrm{Cl}((G,\Lambda )))].$$

Set (R,Λ)=Int(Cl((G,Λ))) is soft regular open and $\left(D,\Lambda )=(G,\Lambda )\overset{˜}{\cup }(\overset{˜}{Z}﹨\mathrm{Cl}((G,\Lambda ))\right)$ is soft dense in $\overset{˜}{Z}$, and thus $\left(G,\Lambda )=(R,\Lambda )\overset{˜}{\cap }(D,\Lambda \right)$.

Conversely, if $\left(R,\Lambda )\overset{˜}{\cap }(D,\Lambda )=(G,\Lambda \right)$, where (R,Λ) is soft regular open and (D,Λ) is soft dense in $\overset{˜}{Z}$. Since (Z,T,Λ) is soft submaximal, so (D,Λ) is soft open and hence (G,Λ) is soft open as it is a union of two soft open sets. □

## 4 Characterizations of soft submaximal spaces

Lemma 4.1 *Let* (Y,Λ) *be a soft subset of* (Z,T,Λ) *. Then* (Y,Λ) *is soft preopen if and only if* $\left(Y,\Lambda )=(U,\Lambda )\overset{˜}{\cap }(D,\Lambda \right)$ *, where* (U,Λ) *is soft open and* (D,Λ) *is soft dense over Z.*

Proof If $\left(Y,\Lambda )\overset{˜}{\subseteq }\mathrm{Int}(\mathrm{Cl}((Y,\Lambda ))\right)$, then (U,Λ)=Int(Cl((Y,Λ))) is soft open. Set $\left(D,\Lambda )=\overset{˜}{Z}﹨(U,\Lambda )﹨(Y,\Lambda )=[\overset{˜}{Z}﹨(U,\Lambda )]\overset{˜}{\cup }(Y,\Lambda \right)$. Now,

$$\overset{˜}{Z}=\mathrm{Cl}((Y,\Lambda ))\overset{˜}{\cup }[\overset{˜}{Z}﹨\mathrm{Cl}((Y,\Lambda ))]=\mathrm{Cl}((Y,\Lambda ))\overset{˜}{\cup }[\overset{˜}{Z}﹨(U,\Lambda )]=\mathrm{Cl}((D,\Lambda )),$$

which concludes that (D,Λ) is soft dense. Thus $\left(Y,\Lambda )=(U,\Lambda )\overset{˜}{\cap }(D,\Lambda \right)$.

Conversely, if $\left(Y,\Lambda )=(U,\Lambda )\overset{˜}{\cap }(D,\Lambda \right)$, for some soft open (U,Λ) and soft dense (D,Λ), then $\left(Y,\Lambda )\overset{˜}{\subseteq }(U,\Lambda )\overset{˜}{\subseteq }\mathrm{Int}(\mathrm{Cl}((U,\Lambda )))=\mathrm{Int}(\mathrm{Cl}((Y,\Lambda ))\right)$ and so $\left(Y,\Lambda )\overset{˜}{\subseteq }\mathrm{Int}(\mathrm{Cl}((Y,\Lambda ))\right)$. □

Theorem 4.2 *The following statements are equivalent for a soft topological space*(Z,T,Λ)*:*

*(1)* (Z,T,Λ)*is a soft submaximal space;*
*(2)* *Each soft preopen set over Z is soft open.*

Proof (1) ⇒ (2) Let (U,Λ) be soft preopen. By Lemma 4.1, $\left(U,\Lambda )=(G,\Lambda )\overset{˜}{\cap }(D,\Lambda \right)$, for some soft open (G,Λ) and soft dense (D,Λ). By (1) (D,Λ) is soft open and so (U,Λ) is a soft open set. Hence (2).

(2) ⇒ (1) Let (D,Λ) be a soft dense set over *Z*. Then $\left(D,\Lambda )\overset{˜}{\subseteq }\overset{˜}{Z}=\mathrm{Int}(\overset{˜}{Z})=\mathrm{Int}(\mathrm{Cl}((D,\Lambda ))\right)$. Therefore (D,Λ) is soft preopen and by (2) (D,Λ) is soft open. Thus (Z,T,Λ) is soft submaximal. □

We assert that the above conclusion appeared in Theorem 16 [21], but our proof is different.

Theorem 4.3 *The following statements are equivalent for a soft topological space* (Z,T,Λ) *:*

*(1)* (Z,T,Λ) *is soft submaximal;*
*(2)* *Each soft subset in* $\overset{˜}{Z}$ *is soft locally closed;*
*(3)* *Each soft subset in* $\overset{˜}{Z}$ *is soft co-locally closed.*

Proof (1) ⇒ (2) Given a soft set (Y,Λ) over *Z*. Since $\mathrm{Cl}((Y,\Lambda ))\overset{˜}{\subseteq }\overset{˜}{Z}$, by Proposition 3.2, Cl((Y,Λ)) is soft submaximal. Since (Y,Λ) is soft dense in Cl((Y,Λ)), so (Y,Λ) is soft open in Cl((Y,Λ)). Therefore $\left(Y,\Lambda )=(G,\Lambda )\overset{˜}{\cap }\mathrm{Cl}((Y,\Lambda )\right)$ for some soft open (G,Λ) over *Z*. Hence (Y,Λ) is a soft locally closed set.

(2) ⇒ (1) Let (D,Λ) be a soft dense in $\overset{˜}{Z}$. By (2) $\left(D,\Lambda )=(G,\Lambda )\overset{˜}{\cap }(F,\Lambda \right)$ for some soft open (G,Λ) and soft closed (F,Λ). We obtain $\left(D,\Lambda )\overset{˜}{\subseteq }(F,\Lambda \right)$ and then $\overset{˜}{Z}=\mathrm{Cl}((D,\Lambda ))\overset{˜}{\subseteq }(F,\Lambda )$. This implies that $(F,\Lambda )=\overset{˜}{Z}$ and so (D,Λ)=(G,Λ) which is a soft open set over *Z*. This proves (1).

(2) ⟺ (3) They are complementary of each other. □

Theorem 4.4 *The following statements are equivalent for a soft topological space* (Z,T,Λ) *:*

*(1)* (Z,T,Λ) *is soft submaximal;*
*(2)* *The soft boundary of any soft set over Z is soft discrete;*
*(3)* *The soft boundary of any soft set over Z is soft closed;*
*(4)* *Each soft co-dense set over Z is soft closed.*

Proof (1) ⇒ (2) Let (Y,Λ) be a soft set. Let $z(e)\overset{˜}{\in }{\mathrm{Cl}}_{T}((Y,\Lambda ))\overset{˜}{\cap }{\mathrm{Cl}}_{T}({\left(Y,\Lambda \right)}^{c})$. By Proposition 3.2, ${\mathrm{Cl}}_{T}((Y,\Lambda )),{\mathrm{Cl}}_{T}({\left(Y,\Lambda \right)}^{c})$ are soft submaximal subspaces of $\overset{˜}{Z}$. Since (Y,Λ) is soft dense in ${\mathrm{Cl}}_{T}((Y,\Lambda ))$, also $\left(Y,\Lambda )\overset{˜}{\cup }\{z(e)\right\}$ is soft dense in ${\mathrm{Cl}}_{T}((Y,\Lambda ))$. By soft submaximality of ${\mathrm{Cl}}_{T}((Y,\Lambda ))$, there is soft open (G,Λ) over *Z* such that $\left(Y,\Lambda )\overset{˜}{\cup }\{z(e)\}={\mathrm{Cl}}_{T}((Y,\Lambda ))\overset{˜}{\cap }(G,\Lambda \right)$. By the same reasoning, we can have ${\left(Y,\Lambda \right)}^{c}\overset{˜}{\cup }\{z(e)\}={\mathrm{Cl}}_{T}({\left(Y,\Lambda \right)}^{c})\overset{˜}{\cap }(H,\Lambda )$ for some soft open set (H,Λ) over *Z*. Therefore

$$\{z(e)\}=(Y,\Lambda )\overset{˜}{\cup }\{z(e)\}\overset{˜}{⋂}{\left(Y,\Lambda \right)}^{c}\overset{˜}{\cup }\{z(e)\}={\mathrm{Cl}}_{T}((Y,\Lambda ))\overset{˜}{\cap }(G,\Lambda )\overset{˜}{⋂}{\mathrm{Cl}}_{T}({\left(Y,\Lambda \right)}^{c})\overset{˜}{\cap }(H,\Lambda )={\mathrm{Cl}}_{T}((Y,\Lambda ))\overset{˜}{\cap }{\mathrm{Cl}}_{T}({\left(Y,\Lambda \right)}^{c})\overset{˜}{⋂}(G,\Lambda )\overset{˜}{\cap }(H,\Lambda )=\mathrm{Bd}((Y,\Lambda ))\overset{˜}{⋂}(G,\Lambda )\overset{˜}{\cap }(H,\Lambda ).$$

Thus {z(e)} is a soft open subset of Bd((Y,Λ)) and so Bd((Y,Λ)) is soft discrete.

(2) ⇒ (3) Let (Y,Λ) be a soft set. By (2) Bd((Y,Λ)) is soft discrete, so Bd((Y,Λ)) has no soft limit point. Hence Bd((Y,Λ)) is soft closed.

(3) ⇒ (4) Given a soft set (Y,Λ), if (Y,Λ) is soft co-dense, $\left(Y,\Lambda )\overset{˜}{\subseteq }\mathrm{Cl}((Y,\Lambda ))=\mathrm{Bd}((Y,\Lambda )\right)$. Thus (Y,Λ) is soft closed.

(4) ⇒ (1) The complement of (4) is (1). The result is proved. □

Definition 4.5 [8] Let (Z,T,Λ) be a soft topological space and let $\overset{˜}{\Phi }\neq (Y,\Lambda )\overset{˜}{\in }T$. The family $T\overset{˜}{\cup }\{(Y,\Lambda )\}$ generates a soft topology $T^{⁎}$ on *Z* called a simple extension of T via (Y,Λ). We shall denote $T^{⁎}=T[(Y,\Lambda )]$ (or briefly, $T^{⁎}=T[Y]$).

Lemma 4.6 *[8, Remark 2.2]* *Let* $T^{⁎}=T[Y]$ *be a simple extension of a soft topological space* (Z,T,Λ) *. Then*

$${\mathrm{Cl}}_{T^{⁎}}((X,\Lambda ))={\mathrm{Cl}}_{T}((X,\Lambda ))\overset{˜}{⋂}\left[{\left(X,\Lambda \right)}^{c}\overset{˜}{⋃}{\mathrm{Cl}}_{Y}((X,\Lambda )\overset{˜}{⋂}(Y,\Lambda ))\right]$$

*for each soft set* (X,Λ) *over Z.*

Lemma 4.7 *Let* $T^{⁎}=T[Y]$ *be a simple extension of a soft topological space* (Z,T,Λ) *. If* (Y,Λ) *is a soft* T *-dense set, then it is soft* $T^{⁎}$ *-dense.*

Proof Apply Lemma 4.6. □

Lemma 4.8 *Let* $T^{⁎}=T[Y]$ *be a simple extension of a soft topological space* (Z,T,Λ) *. If* (Y,Λ) *is a soft* T *-dense set, then*

*(1)* *If* (U,Λ) *is a soft* $T^{⁎}$ *-open set,* ${\mathrm{Cl}}_{T}((U,\Lambda ))={\mathrm{Cl}}_{T^{⁎}}((U,\Lambda ))$ *.*
*(2)* *If* (F,Λ) *is a soft* $T^{⁎}$ *-closed set,* ${\mathrm{Int}}_{T}((F,\Lambda ))={\mathrm{Int}}_{T^{⁎}}((F,\Lambda ))$ *.*
*(3)* *If* (U,Λ) *is a soft* $T^{⁎}$ *-open set,* ${\mathrm{Int}}_{T}({\mathrm{Cl}}_{T}((U,\Lambda )))={\mathrm{Int}}_{T^{⁎}}({\mathrm{Cl}}_{T^{⁎}}((U,\Lambda )))$ *.*
*(4)* *If* (U,Λ) *is a soft* $T^{⁎}$ *-open set, then* (U,Λ) *is a soft regular* T *-open if and only if it is soft regular* $T^{⁎}$ *-open.*

Proof (1) Let (U,Λ) be a soft $T^{⁎}$-open set. Since $T\overset{˜}{\subset }T^{⁎}$, we consider two cases: (a) If $(U,\Lambda )\overset{˜}{\in }T$, clearly ${\mathrm{Cl}}_{T^{⁎}}((U,\Lambda ))\overset{˜}{\subseteq }{\mathrm{Cl}}_{T}((U,\Lambda ))$. On the other hand, suppose $z(e)\overset{˜}{\in }{\mathrm{Cl}}_{T}((U,\Lambda ))$. Let (V,Λ) be any soft $T^{⁎}$-open set containing z(e). There exist soft T-open sets (G,Λ) and (H,Λ) such that $\left(V,\Lambda )=(G,\Lambda )\overset{˜}{\cup }[(H,\Lambda )\overset{˜}{\cap }(Y,\Lambda )\right]$. If $z(e)\overset{˜}{\in }(G,\Lambda )$, then $(G,\Lambda )\overset{˜}{\cap }(U,\Lambda )\neq \overset{˜}{\Phi }$ and so $(V,\Lambda )\overset{˜}{\cap }(U,\Lambda )\neq \overset{˜}{\Phi }$. If $z(e)\overset{˜}{\in }(H,\Lambda )\overset{˜}{\cap }(Y,\Lambda )$, then $(H,\Lambda )\overset{˜}{\cap }(U,\Lambda )\neq \overset{˜}{\Phi }$. Since $\left(H,\Lambda )\overset{˜}{\cap }(U,\Lambda \right)$ is non-null soft T-open and (Y,Λ) is soft T-dense, so $(H,\Lambda )\overset{˜}{\cap }(U,\Lambda )\overset{˜}{\cap }(Y,\Lambda )\neq \overset{˜}{\Phi }$. This implies that $(V,\Lambda )\overset{˜}{\cap }(U,\Lambda )\neq \overset{˜}{\Phi }$ and thus $z(e)\overset{˜}{\in }{\mathrm{Cl}}_{T^{⁎}}((U,\Lambda ))$. Hence ${\mathrm{Cl}}_{T}((U,\Lambda ))={\mathrm{Cl}}_{T^{⁎}}((U,\Lambda ))$.

(b) If $(U,\Lambda )\overset{˜}{\in }T^{⁎}$, then $\left(U,\Lambda )=(G,\Lambda )\overset{˜}{\cup }[(H,\Lambda )\overset{˜}{\cap }(Y,\Lambda )\right]$ for some soft T-open sets (G,Λ),(H,Λ). By (a) we have ${\mathrm{Cl}}_{T}((G,\Lambda ))={\mathrm{Cl}}_{T^{⁎}}((G,\Lambda ))$. Since $T\overset{˜}{\subset }T^{⁎}$, (H,Λ) is also soft $T^{⁎}$-open. Evidently, $\left(H,\Lambda )\overset{˜}{\cap }(Y,\Lambda \right)$ is soft T-dense because (Y,Λ) is soft T-dense. Therefore, by Lemma 4.7, we obtain that

$${\mathrm{Cl}}_{T^{⁎}}((H,\Lambda )\overset{˜}{\cap }(Y,\Lambda ))={\mathrm{Cl}}_{T^{⁎}}((H,\Lambda ))={\mathrm{Cl}}_{T}((H,\Lambda ))={\mathrm{Cl}}_{T}((H,\Lambda )\overset{˜}{\cap }(Y,\Lambda )).$$

In conclusion,

$${\mathrm{Cl}}_{T^{⁎}}((U,\Lambda ))={\mathrm{Cl}}_{T^{⁎}}((G,\Lambda ))\overset{˜}{⋃}{\mathrm{Cl}}_{T^{⁎}}((H,\Lambda )\overset{˜}{\cap }(Y,\Lambda ))={\mathrm{Cl}}_{T}((G,\Lambda ))\overset{˜}{⋃}{\mathrm{Cl}}_{T}((H,\Lambda )\overset{˜}{\cap }(Y,\Lambda ))={\mathrm{Cl}}_{T}((U,\Lambda )).$$

(2) By part (1) and Lemma 2.6.

Also, the other parts can be followed from (1) and (2). □

Theorem 4.9 *Let* (Z,T,Λ) *be a soft topological space. The following statements are equivalent:*

*(1)* (Z,T,Λ) *is soft submaximal;*
*(2)* *If each soft regular* $T^{⁎}$ *-open set is soft* T *-open for any s-extension* $T^{⁎}=T[Y]$ *of* T *via* (Y,Λ) *, then* $T=T^{⁎}$ *;*
*(3)* *If the families of all soft regular* $T^{⁎}$ *-open and all soft regular* T *-open sets over Z are identical for any s-extension* $T^{⁎}=T[Y]$ *of* T *via* (Y,Λ) *, then* $T=T^{⁎}$ *.*

Proof (1) ⇒ (2) We only need to show that $T^{⁎}\overset{˜}{\subseteq }T$ as the reverse is always true. Let $(G,\Lambda )\in T^{⁎}$. Then ${\mathrm{Int}}_{T^{⁎}}({\mathrm{Cl}}_{T^{⁎}}((G,\Lambda )))=(H,\Lambda )$ is soft regular $T^{⁎}$-open. By assumption, (H,Λ) is soft T-open. But $\left(G,\Lambda )\overset{˜}{\subseteq }{\mathrm{Int}}_{T^{⁎}}({\mathrm{Cl}}_{T^{⁎}}((G,\Lambda )))=(H,\Lambda )\overset{˜}{\subseteq }({\mathrm{Cl}}_{T^{⁎}}((G,\Lambda ))\overset{˜}{\subseteq }({\mathrm{Cl}}_{T}((G,\Lambda )\right)$ which means that (G,Λ) is soft T-dense in (H,Λ). Since (Z,T,Λ) is soft submaximal, by Proposition 3.2, (H,Λ) is a soft submaximal subspace, and so (G,Λ) is soft $T_{H}$-open. Since (H,Λ) is soft T-open, by Theorem 2 in [30], (G,Λ) is soft T-open and hence $T=T^{⁎}$.

(2) ⇒ (3) Straightforward.

(3) ⇒ (1) Let (Y,Λ) be a soft T-dense set. Since $T\overset{˜}{\subseteq }T^{⁎}$, by Lemma 4.8, soft regular $T^{⁎}$-open sets over *Z* are equal to soft regular T-open sets and then, by (3), $T=T^{⁎}$. Hence (Y,Λ) must be soft T-open. Thus (Z,T,Λ) is soft submaximal. □

## 5 Conclusion

After the work of Shabir and Naz [30], several types of soft topological spaces have been analyzed. For example: soft compact, soft connected, soft paracompact, soft extremally disconnected, soft separable, soft $T_{i}$-spaces, i=0,1,…,4, and so on. We have continued working in the same direction by studying the class of soft submaximal spaces. We have shown any soft subspace of a soft submaximal space is soft submaximal. The soft product of two soft submaximal spaces need not be soft submaximal. The soft sum of any collection of soft submaximal spaces is soft submaximal space. The soft submaximal spaces are preserved under soft open surjections. Furthermore, we have described soft submaximal spaces in terms of different forms of soft sets.

## Declarations

### Author contribution statement

**Samer Al Ghour**, **Zanyar A. Ameen:** Conceived and designed the experiments; Performed the experiments; Analyzed and interpreted the data; Contributed reagents, materials, analysis tools or data; Wrote the paper.

### Funding statement

This research did not receive any specific grant from funding agencies in the public, commercial, or not-for-profit sectors.

### Data availability statement

No data was used for the research described in the article.

### Declaration of interests statement

The authors declare no conflict of interest.

### Additional information

No additional information is available for this paper.
